# A Comparative Review of Meshed Versus Unmeshed Grafts in Split-Thickness Skin Grafting: Clinical Implications and Outcomes

**DOI:** 10.7759/cureus.69606

**Published:** 2024-09-17

**Authors:** Joben Samuel, Pankaj Gharde, Dheeraj Surya, Shubham Durge, Vasundara Gopalan

**Affiliations:** 1 General Surgery, Jawaharlal Nehru Medical College, Datta Meghe Institute of Higher Education and Research, Wardha, IND

**Keywords:** aesthetic outcomes, graft survival, meshed grafts, split-thickness skin grafting, surgical complications, unmeshed grafts

## Abstract

Split-thickness skin grafting (STSG) is a cornerstone technique in reconstructive surgery, offering solutions for covering wounds, burns, and other skin defects. This review compares meshed versus unmeshed STSG, focusing on their clinical implications and outcomes. Meshed grafts, created by perforating the skin graft to form a mesh-like pattern, are frequently used for larger or irregularly shaped areas due to their ability to expand and conform to the underlying tissue. In contrast, unmeshed grafts are applied as whole sheets, making them suitable for smaller or cosmetically sensitive regions where appearance is paramount. This review examines various aspects of these graft types, including graft survival rates, aesthetic and functional results, healing times, and complications such as infection and graft contraction. This study aims to identify the relative advantages and drawbacks of meshed versus unmeshed grafts by analyzing data from clinical trials, meta-analyses, and systematic reviews. The findings highlight that while meshed grafts offer improved coverage and reduced risk of graft failure, unmeshed grafts are preferred for their superior cosmetic outcomes. Understanding these differences is crucial for optimizing surgical strategies and improving patient outcomes. The review also addresses patient-specific factors and recommends selecting the appropriate graft type based on clinical scenarios.

## Introduction and background

Split-thickness skin grafting (STSG) is a critical technique in reconstructive and dermatologic surgery, utilized to cover wounds, burns, and other skin defects by transplanting a thin layer of skin from a donor site to a recipient site [1]. This procedure involves harvesting the epidermis and a portion of the dermis, which are then applied to the affected area to support healing and restore skin integrity. The primary aim of STSG is to achieve functional and aesthetic restoration of the damaged skin, enhancing both patient quality of life and overall clinical outcomes [1].

The choice between meshed and unmeshed grafts plays a significant role in determining the success of STSG. Meshed grafts are created using a specialized device that perforates the graft in a mesh-like pattern, allowing it to stretch and adapt to irregularly shaped or larger areas [2]. This technique promotes better coverage and reduces the likelihood of graft failure. In contrast, unmeshed grafts are applied as whole sheets of skin without any perforations, typically used for smaller or cosmetically sensitive areas where appearance is a major concern. The type of graft selected can substantially impact various clinical outcomes, including graft survival, aesthetic results, and patient satisfaction, making it crucial to understand the differences between these graft types [3].

This review aims to comprehensively compare meshed versus unmeshed split-thickness skin grafts by examining their clinical outcomes and implications. The first objective is to compare the clinical outcomes associated with each type of graft, focusing on factors such as graft survival rates, aesthetic results, and functional improvements. By analyzing these outcomes, the review seeks to identify which graft type provides superior performance and better meets clinical goals. The second objective is to evaluate the efficacy, complications, and overall patient outcomes related to meshed and unmeshed grafts. This includes assessing healing times, wound closure rates, and complication rates, as well as considering patient-specific factors that may influence the success of each graft type. By addressing these objectives, the review aims to provide valuable insights that can guide clinicians in choosing the most appropriate graft type for different clinical scenarios, ultimately enhancing surgical outcomes and patient care.

## Review

Overview of split-thickness skin grafting

**Figure 1:**
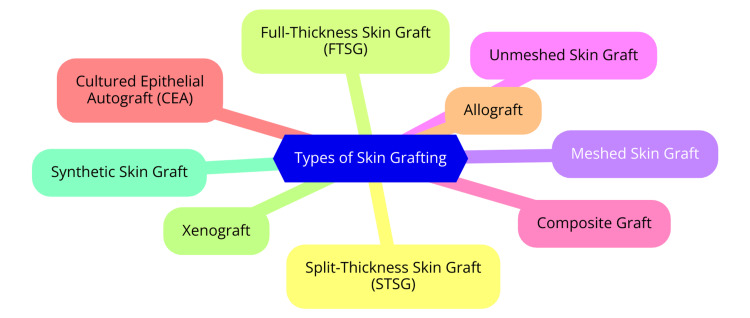
Types of skin grafting Image credit: Dr. Vasundara Gopalan

STSG is a commonly employed surgical technique to cover wounds and skin defects by transplanting a section of skin that includes the epidermis and a superficial layer of the dermis [1]. This technique is particularly effective in reconstructive surgery for treating a variety of conditions, such as burns, skin cancer excisions, and traumatic injuries. The grafts are typically thin, with thickness ranging from 0.125 mm to 0.75 mm, and are classified into thin, medium, and thick grafts based on their thickness [1]. The harvesting of STSGs is generally performed with a dermatome, which may be either air-powered or electric. This specialized device facilitates the rapid and uniform harvesting of grafts, with adjustable thickness to meet surgical requirements. In some cases, manual techniques using a scalpel may still be used. Anesthesia reduces discomfort during the procedure, enhancing patient comfort and procedural efficiency [4]. There are two primary types of STSGs: meshed and unmeshed. Meshed grafts are created by cutting a grid-like pattern into the graft, allowing it to expand and cover a larger wound area. This meshing technique aids in fluid drainage, reducing the risk of complications such as seroma or hematoma beneath the graft. Advantages of meshed grafts include the ability to cover a larger surface area from a limited donor site and easier application to larger defects. However, they may have aesthetic drawbacks due to their "fishnet" appearance. They generally require a longer re-epithelialization period, as keratinocytes must proliferate from the graft edges to fill the mesh gaps [1]. In contrast, unmeshed grafts, also known as sheet grafts, are harvested without meshing and are typically used when sufficient donor skin is available. These grafts provide a continuous layer of skin, leading to faster re-epithelialization than meshed grafts [1]. Additionally, unmeshed grafts offer a more aesthetically pleasing appearance, with a uniform skin surface and greater resilience to movement, as they are less fragile than meshed grafts. However, they require a larger amount of donor skin, which may not always be available, and carry a higher risk of fluid accumulation under the graft due to the lack of drainage [1]. Types of skin grafting are shown in Figure 1.

Clinical outcomes

Evaluating the clinical outcomes of meshed versus unmeshed STSGs involves several critical factors, including graft take and survival rates, aesthetic and functional results, and healing time. A thorough understanding of these aspects enables clinicians to make well-informed decisions tailored to each patient's needs [2]. When comparing the survival rates of meshed and unmeshed grafts, meshed STSGs typically exhibit graft take rates ranging from 80% to 95%. However, the survival rate can be affected by the expansion ratio used during graft preparation; higher ratios may reduce graft survival due to increased fragility [5]. Conversely, unmeshed grafts generally demonstrate higher survival rates, often exceeding 95%. This is due to their continuous structure, which promotes better vascularization and reduces the risk of complications such as seroma formation [3]. Several factors influence graft take, including the donor site's condition and the recipient site's characteristics. A well-vascularized donor site is essential for optimal graft viability [3]. Additionally, the health of the recipient site, including the presence of infection and the quality of wound bed preparation, significantly impacts graft success. Graft thickness also plays a role, thicker grafts tend to have higher survival rates but may increase the likelihood of complications like delayed healing. Effective postoperative care, including proper immobilization, infection monitoring, and exudate management, is crucial for improving graft survival [6]. The aesthetic outcomes of meshed and unmeshed grafts can differ significantly. Meshed grafts, while effective for covering larger areas, often result in more pronounced scarring due to the grid-like pattern created during the meshing process. Despite this, they can still offer satisfactory cosmetic results, especially when donor skin is limited. In contrast, unmeshed grafts typically produce superior cosmetic outcomes, characterized by smoother and less noticeable scars. Patients often report higher satisfaction with the aesthetic results of unmeshed grafts, making them a preferred choice in many situations [7].

Regarding functional outcomes, both graft types can impact a range of motion, particularly near joints. However, unmeshed grafts generally allow for better mobility post-healing due to their smoother surface and reduced scarring. Sensory recovery may also vary; studies suggest that unmeshed grafts may provide better sensory outcomes, as their complete structure can facilitate more effective nerve regeneration [8]. Healing time and wound closure are crucial when comparing meshed and unmeshed grafts. Meshed grafts typically require two to four weeks of healing, depending on the mesh size and the patient’s overall health. Re-epithelialization may take longer due to the gaps created by meshing, which can be a disadvantage in acute situations where rapid closure is desired. In contrast, unmeshed grafts usually heal faster, with complete healing occurring in one to three weeks. The continuous nature of unmeshed grafts facilitates quicker re-epithelialization, reducing overall healing time [1]. For wound closure, meshed grafts may take longer due to the need for epithelial cells to migrate across the mesh openings. This extended healing time can increase the risk of complications related to prolonged exposure to the wound bed. On the other hand, unmeshed grafts generally achieve faster wound closure, providing an advantage in overall healing time and minimizing the risk of complications [9].

Complications and adverse effects

When comparing meshed and unmeshed STSGs, various complications and adverse effects can arise, particularly concerning infection rates, graft contraction, scar formation, and other graft-related issues. Infection incidence significantly impacts the overall success of skin graft procedures [1]. Research suggests that meshed grafts may exhibit a lower infection rate due to their ability to effectively drain exudates, thus reducing the risk of seroma and hematoma formation [10]. For instance, a study involving meshed STSGs for scrotal skin defects reported graft loss. However, infection rates were not explicitly detailed, indicating that while complications occurred, they were manageable without requiring additional surgeries [2]. Conversely, unmeshed grafts, though providing superior aesthetic results, may be at a higher risk for infection due to fluid accumulation beneath the graft, which creates a conducive environment for bacterial growth [9]. Graft contraction and scar quality are crucial factors affecting both the aesthetic and functional outcomes of skin grafting. Meshed grafts tend to heal with more noticeable scarring due to the secondary healing process through the mesh's interstices. This can lead to more prominent scars than unmeshed grafts, which generally offer a smoother and more uniform appearance [1]. Studies have indicated that while meshed grafts are useful for covering larger areas and beneficial in cases with limited donor skin, they may produce less favorable cosmetic results than sheet grafts. In contrast, unmeshed grafts yield better cosmetic outcomes and lower contracture rates, making them more suitable for cosmetically sensitive areas [11]. Additional complications associated with skin grafts include seroma and hematoma formation. Meshing the graft facilitates better fluid drainage, which reduces the likelihood of these complications, particularly in larger grafts or areas prone to significant fluid accumulation. On the other hand, unmeshed grafts are more susceptible to seroma and hematoma due to the absence of drainage holes, potentially leading to graft failure if not managed appropriately [1]. The complications and adverse effects of meshed versus unmeshed grafts in STSG are summarized in Table 1.

**Table 1:** Complications and adverse effects of meshed versus unmeshed grafts in STSG STSG: split-thickness skin grafting

Complication/adverse effect	Meshed graft	Unmeshed graft	Clinical relevance
Infection rate [12]	Higher due to increased wound exposure	Lower due to intact skin coverage	Infection control is critical for wound healing
Graft failure (necrosis) [1]	Increased risk with larger mesh sizes	Lower risk due to better vascularization	Critical for graft success and healing
Scarring [13]	Higher scarring risk due to mesh pattern	Lower risk with more uniform healing	Aesthetic outcome is important in visible areas
Contracture formation [14]	Greater risk due to mesh contraction	Lower risk	This can lead to functional limitations
Fluid loss [15]	Higher due to open spaces in the graft	Lower due to complete skin coverage	Important in large surface area burns
Pain [16]	Moderate	Lower	Affects patient comfort and recovery
Re-epithelialization time [17]	Faster, due to a larger surface area	Slower, more controlled	Impacts overall healing time
Cosmetic outcome [18]	Poorer cosmetic appearance	Better cosmetic appearance	Critical for areas like the face or hands
Donor site morbidity [19]	More frequent, depending on mesh size	Less frequent	Affects recovery and donor site healing

Patient and surgical factors influencing outcomes

A range of patient demographics and surgical factors influences the outcomes of skin grafting procedures. Understanding these variables is crucial for optimizing surgical techniques and enhancing patient care [20]. Age is a significant predictor of morbidity and mortality in patients undergoing skin grafting, particularly among burn victims. Advanced age is often associated with increased comorbidities, such as diabetes mellitus and hypertension, which can impair healing and elevate the risk of graft loss. Research suggests older adults experience higher graft failure rates due to physiological changes and diminished physiological reserves [21]. Comorbidities also play a critical role in determining the success of skin grafts. Chronic conditions such as congestive heart failure (CHF) and diabetes are linked to higher graft failure rates. For example, patients with CHF are approximately 2.55 times more likely to experience graft failure compared to those without this condition. Other comorbidities, including peripheral vascular disease and obesity, also contribute to poorer outcomes in skin grafting by hindering blood flow and overall healing [22]. Skin characteristics, including elasticity and thickness, can impact graft acceptance and healing. Patients with certain skin types may experience different rates of graft contraction and pigmentation changes post-surgery, affecting both the aesthetic and functional outcomes of the graft [23]. The donor site characteristics significantly affect graft outcomes as well. Sites with good vascularity and minimal scarring generally yield better grafts, as they provide a more favorable environment for healing. Additionally, the choice of donor site impacts the healing process and the overall aesthetic result of the graft. Selecting an optimal donor site is critical for maximizing the success of the procedure [24]. The surgical technique used for harvesting and applying the graft is another vital factor in the success of skin grafting. For instance, meshed grafts, while allowing for expansion and drainage, may result in slower re-epithelialization than unmeshed grafts. The choice between meshed and unmeshed techniques should be tailored to the specific clinical scenario and patient needs, considering the wound characteristics and desired outcomes [1]. Finally, the size and location of the graft play a significant role in determining outcomes. Larger grafts and those placed in high-movement areas, such as joints, may have higher failure rates due to shear forces and tension. Studies indicate that graft location influences healing times and the risk of complications like seroma formation and infection. Thus, careful graft size and location planning are essential for enhancing the likelihood of successful healing and minimizing complications [25]. Patient and surgical factors influencing outcomes in meshed versus unmeshed grafts in STSG are summarized in Table 2.

**Table 2:** Patient and surgical factors influencing outcomes in meshed versus unmeshed grafts in STSG STSG: split-thickness skin grafting

Factor	Meshed graft	Unmeshed graft	Impact on outcome
Patient age [26]	Better for elderly patients due to rapid epithelialization	Suitable for younger patients with better healing capacity	Age influences healing speed and tissue recovery
Nutritional status [27]	Malnourished patients may experience delayed healing	May perform better with adequate nutrition	Nutrition plays a vital role in wound healing
Wound size [28]	More effective for large wounds	Suitable for smaller wounds	Larger wounds may benefit from mesh expansion
Wound depth [9]	Suitable for shallow and intermediate wounds	Better for superficial wounds	Depth influences graft take and integration
Immune status [29]	Immunocompromised patients may experience more complications	Less risky for immunocompromised patients	Immune function affects infection rates and healing
Diabetes or chronic conditions [30]	Higher risk of complications (e.g., infection)	Less risk but slower healing	Diabetes and chronic conditions affect overall outcomes
Burn severity [31]	Optimal for severe burns requiring rapid coverage	Better for minor burns with less risk of contraction	Severity dictates the choice of graft type
Surgical technique [32]	Requires precision in spreading and fixing	Simpler to apply and fix	Surgeon’s skill affects graft take and healing
Donor site condition [33]	Suitable for limited donor sites	Requires healthy donor site for larger unmeshed grafts	Donor site health influences graft success
Postoperative care compliance [34]	Requires vigilant care due to higher risk of infection and complications	Easier postoperative care	Patient adherence to care can improve outcomes

Comparative studies and evidence

Several key clinical trials have examined the differences between meshed and unmeshed STSGs, offering valuable insights into their outcomes. Comparative studies and evidence regarding meshed versus unmeshed STSGs are summarized in Table 3.

**Table 3:** Comparative studies and evidence on meshed versus unmeshed STSG STSG: split-thickness skin grafts; NPWT: negative pressure wound therapy

Authors	Year	Meshed vs. unmeshed	Main findings
Black et al. [35]	2004	Meshed (1:1) vs. unmeshed	100% graft take with meshed grafts; good cosmetic and functional outcomes
Bonaventura et al. [36]	2021	Meshed vs. unmeshed (delayed procedure)	Single-session meshed grafts resulted in faster healing and fewer bandage applications
Tarar et al. [37]	2016	Transverse meshed vs. unmeshed	Transverse meshing improved graft takes while maintaining a good cosmetic appearance
Sweitzer et al. [38]	2019	Meshed vs. unmeshed with NPWT	No significant difference in graft take or complication rate between meshed and unmeshed grafts with NPWT
Nikkhah et al. [11]	2015	Meshed (1:1) vs. unmeshed	1:1 meshed grafts had slightly better graft survival and similar cosmetic outcomes compared to unmeshed

Cost-effectiveness and resource utilization

The cost implications of using meshed versus unmeshed STSGs can differ substantially due to several factors, including the surgical procedure, materials used, and postoperative care requirements. Meshed grafts typically involve more complex surgical techniques and specialized equipment for meshing, which can increase initial surgical costs compared to unmeshed grafts, which are simpler to apply [1]. Although the cost of the grafts may not vary significantly, meshed grafts often necessitate additional materials, such as dressings and fixation devices, due to their fragility and the need for meticulous postoperative management. Despite the higher initial costs associated with meshed grafts, they may offer potential savings in the long term by reducing complications like graft failure or the need for additional surgeries. Research suggests that meshed grafts may improve overall satisfaction and functional outcomes, indirectly lowering costs related to follow-up care and additional treatments [39]. Meshed grafts can result in shorter hospital stays because they can cover larger areas and facilitate better fluid drainage, which helps reduce complications. In contrast, unmeshed grafts may lead to longer hospitalizations because of potential fluid accumulation beneath the graft, which can cause complications such as seroma or graft loss [40]. Patients with meshed grafts may require fewer follow-up visits for complications related to graft failure, as these grafts generally have better take rates when managed properly. Conversely, unmeshed grafts may necessitate more frequent monitoring due to the risks of fluid retention and graft loss, potentially complicating recovery. Additionally, the need for further treatments, such as revision surgeries or interventions for complications, may be lower with meshed grafts due to their generally superior aesthetic and functional outcomes. While unmeshed grafts may provide better initial cosmetic results, they can lead to higher rates of complications that require additional medical intervention [1].

Future directions and innovations

Recent advancements in skin grafting techniques aim to enhance both the efficiency and effectiveness of grafts. Notable innovations include cultured dermoepithelial autografts, which have advanced to grow entire anatomical structures, such as hands. This development significantly reduces scarring and improves texture, allowing for more comprehensive coverage of skin defects and enhancing aesthetic outcomes [41]. Another promising technology is 3D bioprinting, which enables the creation of skin in large quantities and has shown potential in improving re-epithelialization for full-thickness burns. Although still in developmental stages, 3D printing could automate skin production and increase its accessibility for clinical applications [42]. Additionally, researchers are exploring viral transfection techniques to develop genetically modified grafts that address common skin grafting issues, such as poor vascularization and integration with host tissue. New materials, such as oxygen-degradable polythioketal urethane foams and gelatin-based scaffolds, are also being tested as advanced dermal substitutes. These substitutes reduce inflammation and enhance neo-vascularization, potentially offering more effectiveness than traditional polyester foams [42]. Despite these advancements, several gaps in current research present opportunities for future exploration. A fully functional tissue-engineered skin substitute replicating all aspects of natural skin remains unavailable, and creating a complete skin model that includes all necessary cell types and structures is a critical area for development. Comprehensive studies are needed to evaluate the long-term effectiveness and safety of new grafting technologies, particularly across diverse patient populations and various clinical settings [43]. Research into the role of stem cells in skin grafts could lead to improved healing and functionality, focusing on how these cells enhance graft survival and integration with host tissue. Further investigation into the practical applications of 3D and 4D bioprinting is necessary, including assessing the scalability of production and the viability of printed tissues in human patients [44]. Future directions and innovations in meshed versus unmeshed grafts for STSG are detailed in Table 4.

**Table 4:** Future directions and innovations in meshed versus unmeshed grafts for STSG STSG: split-thickness skin grafting

Innovation/direction	Meshed graft	Unmeshed graft	Potential impact
3D Bioprinting of skin [42]	Potential for custom-designed mesh patterns	Directly applicable for creating full skin layers	Could revolutionize graft customization and reduce healing time
Nanotechnology-enhanced grafts [45]	Could improve infection resistance and integration	It can be used to enhance wound healing and reduce scarring	Enhanced healing and decreased complication rates
Stem cell-infused grafts [46]	Potential to increase healing rates and tissue regeneration	Can promote better integration and reduce donor site morbidity	Accelerates recovery and reduces the need for additional surgeries
Synthetic and bioengineered skin [47]	This may allow for more durable and flexible mesh patterns	Provides an alternative to human grafts for unmeshed applications	Reduces the need for donor skin and expands treatment options
Gene therapy [48]	Could target genes related to wound healing, improving outcomes for meshed grafts	It could help reduce complications and scarring in unmeshed grafts	Customizes treatment based on patient genetics for better results
Advanced wound care technologies [49]	Smart dressings could monitor healing and apply treatments as needed	It can help prevent infection and optimize moisture levels for unmeshed grafts	Improves graft survival and decreases complications
Hybrid grafts [50]	Combining meshed and unmeshed areas for optimized healing in different regions	Allows for selective use in areas requiring higher aesthetic outcomes	Increases flexibility and precision in grafting procedures
AI-assisted surgical techniques [51]	Could improve precision in graft application and reduce human error	Enhances decision-making for graft selection and placement	Optimizes outcomes and reduces surgical time
Regenerative medicine [52]	It may improve the integration of meshed grafts with surrounding tissues	Can improve healing in complex wounds with better tissue regeneration	Potential to reduce recovery time and improve long-term functionality

## Conclusions

In conclusion, choosing between meshed and unmeshed STSGs has significant implications for clinical outcomes and patient care. Both graft types offer distinct advantages and disadvantages, influencing graft survival, aesthetic results, and overall healing. Meshed grafts are often preferred for their ability to effectively cover larger or irregularly shaped areas and reduce graft failure risk. In contrast, unmeshed grafts are chosen for their superior cosmetic results in smaller, more sensitive areas. The review highlights that the optimal choice of graft type should be guided by the specific clinical context, including the size and location of the wound, the desired cosmetic outcome, and the patient's overall health condition. By carefully considering these factors and the associated outcomes of each graft type, clinicians can make more informed decisions, ultimately improving patient satisfaction and surgical success. Continued research and advancements in skin grafting techniques will further refine our understanding of these options, potentially leading to enhanced approaches and better outcomes in reconstructive surgery.
